# Quinine

**Published:** 1860-06

**Authors:** 


[In the absence of the Editor, and the consequent scarcity of Editorial for this number, we introduce the following interesting paper, read by J. N. Graham, M. D., before the Chicago Academy of Medicine.—W. H. B.]

---

### QUININE.—ITS REMEDIAL POWERS.

In presenting a few thoughts on the above agent, I am aware that I shall differ with many Fellows of the Academy who have expressed their views on this subject, and with some learned papers that have been read before us.

Much of the difference of opinion that exists between medical men, on subjects relating to our profession, arises from a misunderstanding of each other's views, or from viewing the same subject from limited or different stand-points.

The army surgeon, in the swamps of Florida, will give quinine with impunity, in 40 to 50 grain doses, without regard to the stage of fever or inflammation—while in the hospitals of Boston, the physician would look with horror upon a five grain dose, and dole out with the greatest precision, as to time, stage, etc., his grain and a half to two grain doses. (This *has* held good there, if not now.)

Now, to a certain extent, both are correct. The large doses necessary to arrest an intermittent in the malarious districts of the South, are not required in the more northern latitudes, where malaria is scarcely known. So in its mode of action upon the physical system—the views of writers are almost as varied as their manner of administration. Dr. Sam'l. Gordon, of Dublin, Ireland, in writing on its pathological effects, quotes Headland to say, "the action of quinia is exerted primarily in the blood and not on the nerves." "Tiedeman and Gmelin found it long ago in the blood of a patient to whom it was administered." Dr. Cochran, in a number of the *Charleston Medical Journal*, in pointing out in what its action consists, says, "In women, under its influence, if they were menstruating, they complained of increase. In some cases it hastened the flow, if given just before the period; it provoked their return when suddenly suppressed by cold, etc." He says also, that "if administered in large doses, and frequently repeated, it defibrinates the blood, rendering it fluid and incoagulable." M. Biquet, of Paris, says "that it does not act on the general state of the organs, or on the blood, but has its special action on the *nervous* system. Dr. H. Bence Jones, F. R. S., says, "Quinine cures neuralgia as it cures ague, by subduing the action of the nerves, not by any *tonic* action." Speaking of its use in typhoid fever, he says, "It is most useful when there is excitement, agitation, excited eye and manner, and delirium." In a learned paper read before the Academy, by our Fellow, Dr. Hamill, it was argued that under the influence of quinine, in malarious fevers, less of urea was excreted by the kidneys, while the waste of the tissue went on, and thus the blood becoming impregnated with urea, there was the greater tendency to delirium—poison, and death. To this it was answered by Prof. Byford, that he believed on the contrary, from his own experience, that quinine, by arresting the disease, *prevented* the waste of tissue and blood derangement, and thus *lessened* the chances for comatism or poison by urea. My own experience would certainly coroborate this view, and it seems to me, that if quinine will cure intermittent, if, as has been abundantly shown, it may be given in grave cases without respect to the stage of fever or congestion, whatever its mode of action, in eliminating the position, it thus cuts short the disease, not by the substitution of "waste of tissue," or blood poison by urea. That such results may and do sometimes follow the use of quinine, is evident, but, from my own experience, I cannot say that I ever saw the slightest reason for attributing them to quinine as the cause. Rather, may we not say, the primary causes at work in *originating* the disease, have, despite of what may have been done, produced the changes in the blood, etc.?

Quinine, in its effects upon the human system, occupies a position that scarcely any other medicinal agent is known to possess. I regard it a *tonic*, a stimulant, a sedative, an anti-periodic, an antispasmodic—a diaphoretic.

"A tonic," says Dr. Eston, Prof. of Mat. Med. in the University of Glasgow, "is a medicine which commencing its action on the stomach itself, very likely by stimulating its nerves and muscular fibres, improves the appetite, strengthens digestion, and feeds assimilation, helps consequently to make good blood, which in turn adds vigor to the circulation, which produces secretion."

In small repeated doses, quinine acts as a *tonic*. In larger, its tonic effect is quickly followed by its stimulating properties, but in grave periodical cases, there exists the most abundant proof that its *sedative* effect is the one which tells most certainly in arresting the disease; to do this it must be given in large repeated doses. This, I have often proven in the treatment of the graver forms of malarious fever in the West and South.

We know, Tartar Emetic in large doses is a powerful remedy against *periodicity*, by its sedative effects; so quinine, without the irritating and prostrating effects of the antimony, seems to hold in subjection the nervous excitement, and by its power over the nerve centres, arrest the paroxysm.

Dr. Holmes, of the U. S. A. Medical Staff in Florida, in writing on the use of large doses of quinine, says, "Quinine as a remedy for periodicity is to be given regardless of any existing state of inflammation."

He recommends never giving it in repeated doses, when directed for the cure of a periodical disease. He says, "In *emergrent* cases it may be given in the lowest state of prostration, or the highest grade of fever."

He says further, "No better diaphoretic than this (quinine) in many stages of the system. When congestion comes on the brain in intermittent and all its collaterals, I have given 40 to 50 grains; have seen its effects on the brain aggravating for the time every symptom, or occasionally but slightly affecting the disease for some hours, then as the sedative effects came on, I have beheld the patient drop into a composed sleep."

In less malarious districts than those referred to by Dr. Holmes, I have often given the quinine in ten to fifteen grain doses, irrespective of fever, delirium, or stupor, and with the happiest effects, the patient sinking from the wildest delirium into profuse perspiration and quiet slumber.

In the more severe forms of intermittent, where the life of the patient seems to hang upon the fact whether he shall have a second or third paroxysm, we have no time to wait for the remission of the fever, or the subsidence of the brain symptoms—the tonic or antiperiodic must be given or the patient dies.

The phenomena attending intermittent fever, leads to the conclusion that the paroxysms are produced by an influence acting through the cerebral centres. Quinine seems to have an affinity (so to speak) for, and exerts a wonderful power in controling or modifying the action of the nervous system. Does it pre-occupy these cerebral centres, and by a power peculiarly its own, expel the morbid influence, and thus cut short—*cure* the disease?

In chorea, in functional epilepsy, hysteriæ and spasmodic asthma, diseases peculiarly of a nervous character, its curative properties have been often signally manifest.

By some, it is contended that it acts as a mere *antiperiodic.* "It is very probable," says Tazwell, of Tennessee, "that it might be profitably employed even where periodicity could not be traced, as according to Prof. Drake, it is a sedative and antispasmodic narcostic." If, as in the definition already given, a tonic is a medicine which improves the appetite, strengthens digestion, and favors assimilation; helps to make good blood, etc., I see not why Dr. Tazwell's views are not correct. Quinine is undeniably a tonic.

I have long been in the habit of giving quinine in small doses, with the object of regulating the appetite, strengthening the system, etc. in cases of slow convalescence from fever, or of general debility from other causes. In some form or other, the quinine enters largely into all my tonic preparations in such cases. The seeming paradox, that the same agent should produce so many different results, and be used under apparently so dissimilar circumstances, must not be argued against the fact that these results are produced. But why not give it during the fever, stupor, or delirium of intermittent? Are not these the very results of the originating cause, be it miasm or what not, poisoning the blood, and thus rendering it powerless to nourish the brain, the very results, which by removing the cause, or rendering it powerless to produce them, the quinine is intended to correct?

Did the cases present, as instanced in the preceding, I would alike give it in the highth of the fever, the wildness of the delirium, or the depths of the stupor, assured of its good effects.

I have never seen quinine cut short typhoid fever, though I have given it in such cases in large doses, and for a purpose; nor have I seen any evil effects from its use in such cases.

The following propositions I think are evident:

1st. Quinine seems to act by the circulation through the cerebral centres, upon the general nervous system.

2d. It is a tonic.

3d. A *Stimulant.*

4th. An *Antiperiodic.*

5th. A Diaphoretic.

6th. An Antispasmodic.

7th. A Narcotic.

8th. These results are produced or modified by *quantity, time, latitude, disease, condition.*

